# Impact of Mechanical Degradation on Polymer Injectivity in Porous Media

**DOI:** 10.3390/polym10070742

**Published:** 2018-07-05

**Authors:** Badar Al-Shakry, Tormod Skauge, Behruz Shaker Shiran, Arne Skauge

**Affiliations:** 1 Department of Chemistry, University of Bergen, Allegaten 41, Bergen 5007, Norway; Arne.Skauge@uib.no; 2 Uni Research CIPR, Allegaten 41, Bergen 5007, Norway; Behruz.Shaker@uni.no; 3 Energy Research Norway, Allegaten 41, Bergen 5007, Norway; Tormod.Skauge@energyresearch.no

**Keywords:** HPAM polymer, rheology, viscosity, injectivity, mechanical degradation, polymer flooding

## Abstract

Polymer flooding is an established enhanced oil recovery (EOR) method; still, many aspects of polymer flooding are not well understood. This study investigates the influence of mechanical degradation on flow properties of polymers in porous media. Mechanical degradation due to high shear forces may occur in the injection well and at the entrance to the porous media. The polymers that give high viscosity yields at a sustainable economic cost are typically large, MW > 10 MDa, and have wide molecular weight distributions. Both MW and the distributions are altered by mechanical degradation, leading to changes in the flow rheology of the polymer. The polymer solutions were subjected to different degrees of pre-shearing and pre-filtering before injected into Bentheimer outcrop sandstone cores. Rheology studies of injected and produced polymer solutions were performed and interpreted together with in situ rheology data. The core floods showed a predominant shear thickening behavior at high flow velocities, which is due to successive contraction/expansion flow in pores. When pre-sheared, shear thickening was reduced but with no significant reduction in in situ viscosity at lower flow rates. This may be explained by reduction in the extensional viscosity. Furthermore, the results show that successive degradation occurred which suggests that the assumption of the highest point of shear that determines mechanical degradation in a porous media does not hold for all field relevant conditions.

## 1. Introduction 

Among several processes which are applied to increase oil recovery, polymer flooding has been widely implemented as a mobility control technique in tertiary enhanced oil recovery (EOR) [1]. The most basic method of recovering oil from a reservoir is by pressure depletion [2]. The pressure difference between the oil reservoir and the surface will lead to production of oil until the reservoir pressure becomes too low for production. Reservoir utilization is poor, typically 95%–80% of the oil remains in the ground, and the energy costs of demobilizing the oil field and remobilizing at a new site is relatively high. The energy recovery is improved by injecting water or gas into the reservoir to maintain pressure. This reduces the remaining oil to 80%–40%. Still, in most cases, there is more oil left in the reservoir than produced at the end of the economic lifetime of the oil field. So-called tertiary methods are used to reduce the remaining oil down to 60%–30% of the initial volume. This includes injection of fluids or gases not naturally present in the reservoir [2]. The purpose of polymer flooding is to improve the sweep efficiency compared to waterflooding by the addition of water-soluble polymer to viscosify the injected fluid. The increase in fluid viscosity results in improved macroscopic displacement of oil by reducing the mobility ratio between the water and the oil phase (injected fluid mobility vs. displaced fluid mobility), which reduces frontal instability. It may also increase microscopic displacement since the viscous force which mobilizes trapped oil droplet may overcome the capillary forces preventing the oil droplets from being mobilized [2,3]. Commonly, two types of polymers have been utilized in EOR applications: synthetic polymers, primarily partially hydrolyzed polyacrylamide (HPAM), and biopolymers, mainly xanthan. Both polymers are used as viscosifying agents [4]. Wang, et al. [5] reported the success of polymer flooding in increasing oil recovery factor of Daqing oil field in China. Among many other successful field polymer flooding projects reported by Standnes and Skjevrak [6], HPAM is the most commonly used EOR polymer.

HPAM transported in oil reservoirs will experience different flow velocities due to high flow rates at the injector and also due to local pore size variations. This results in expansion and contraction of polymer flow inside the porous media. It would accordingly exhibit different flow regimes with respect to shear rates. These different flow regimes have been widely discussed previously, e.g., by Chauveteau [7], Southwick and Manke [8], Stavland, et al. [9], Zamani, et al. [10], and Skauge, et al. [11]. Due to its viscoelastic nature, its in situ viscosity is a contribution of viscous and elastic properties, e.g., shear and extensional viscosity, respectively [12].

In a pure shear flow such as the flow in viscometer, HPAM exhibits shear thinning behavior that can be described by a power law equation [13]. Shear thinning behavior is an ideal injectivity characteristic of EOR polymers, where viscosity decreases with the increase in shear rate. High flow velocities are inherent in wellbore areas [14]. They cause an increase in polymer apparent viscosity in porous medium (shear thickening) in contrast to dominant bulk thinning behavior measured in a viscometer. In a porous medium, at low flow velocities, shear viscosity is dominant while the flow is dominated by extensional viscosity at high velocities [15]. During the extensional flow, polymer coils experience high extensional stresses that induce flow resistance which gives the substantial rise on apparent viscosity [16,17]. This is theoretically interpreted by coil transition theory [7]; however, this theory has been debated subsequently by transient network theory, which explains the origin of shear thickening regarding disentanglement timescale [18,19]. Regardless of the theoretical interpretations of shear thickening, it has been experimentally observed even at very low concentrations of HPAM, see, e.g., [20,21,22,23]. It increases linearly with the flow velocity after the onset of shear thickening [24]. The onset of shear thickening has been given high attention in literature; even more than the effect of the magnitude of shear thickening on viscosity [22]. The onset of shear thickening is a function of many parameters, such as polymer molecular weight, concentration, degree of hydrolysis, salinity, temperature and rock permeability [9,15,23,25].

In conjunction with shear thickening at high flow velocities discussed above, HPAM solution is also prone to mechanical degradation [3,26]. Mechanical degradation of polymer can be described as an irreversible process that leads to the breakage of polymer molecules due to high mechanical stresses induced by high flow velocities or elongational deformations [16,26,27,28]. The breakage of polymer chain induces a significant loss on polymer viscosity. Consequently, it reduces its displacement efficiency [28]. Mechanical degradation is a function of flow velocity, pore geometry, pore tortuosity, polymer-fluid and polymer-rock interactions and physicochemical properties of the polymer. It would be high for high flow rate, high molecular weight polymer, high brine salinity and low formation permeability [26,29]. Claims have been made that mechanical degradation occurs at the entry point of the sand face and therefore is independent of path length [7,28,30,31]. 

Polymer injectivity is strongly bound with its rheology [11,30]. For instance, shear thickening behavior limits polymer injectivity through associative pressure build-up that might cause wellbore fracturing or polymer mechanical degradation. In cases where fracturing or fracture growth occurs due to polymer injection, it might spoil the economy of polymer flooding project due to early breakthrough and loss of polymer sweep efficiency [32]. However, in cases where mechanical degradation occurs, it can alter polymer rheological properties and cause loss in viscosity. Both fracturing and polymer mechanical degradation make the pre-assessment of polymer injectivity challengeable. 

**Figure 1:**
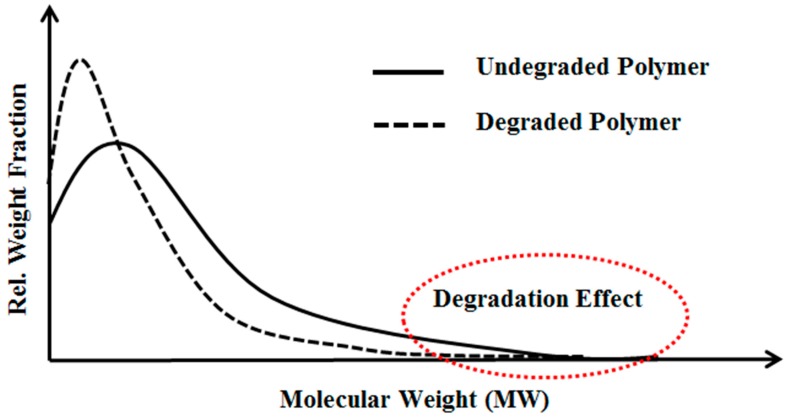
Schematic illustration of the effect of mechanical degradation on polymer molecular weight distribution (MWD) based on the observations reported by Seright, et al. [33]. The peak of the degraded solution shifts to a lower molecular weight. MWD was reduced for the degraded solution.

HPAM mechanical degradation could be minimized if the polymer is submitted for a certain amount of mechanical degradation prior injection into reservoir [2,3,16]. This approach is well-known as the polymer pre-shearing process. This was discussed in Seright, et al. [33], in which mechanical degradation of HPAM solution occurs at the high end of polymer molecular weight distribution (MWD), as shown in Figure 1. This is because high molecular weight molecules have large size, which could offer more resistance to flow. Therefore, large elongational stresses causing breakdown of molecules resulting in degradation. During the pre-shearing process, high molecular weight species will break down into some combination of lower molecular weight fragments, leading to a new MWD. The new MWD of degraded polymer translated into lower MWD. Hence, HPAM viscoelastic properties that depend on high molecular weight species are more affected compared to shear viscosity that depends on average molecular weight which relatively less altered by pre-shearing process [26]. Moreover, the pre-shearing process results in better filterability [34] by removing polymer aggregates or micro gel that responsible for pore blockage. This eliminates the high apparent resistance factors that may appear at low flow rates cause injectivity issues [29]. Chain scission mechanisms associated with polymer degradation or shearing were extensively discussed by Odell, et al. [35] and Muller, et al. [36]. The amount of pre-shearing should be optimized to avoid the loss of polymer viscosity and improve its injectivity characteristics [37]. 

To represent the flow of polymer near wellbore areas and as it advances within the reservoir using linear cores is challenging due to different states of velocity regimes. For example, unsteady state flow conditions are present at wellbore areas, while a steady state condition is applied in the lab for core flood studies. The experimental design of core flood has to consider filtration and degradation effects on polymers, as reported by some earlier studies, e.g., Martin [38,39]. The effect of the prefiltering process in which the polymer subjected to low flow rate before injection has an insignificant impact on polymer in situ viscosity [40]. This is also observed in the experimental study performed by Skauge, et al. [23]. They investigated the role of polymer phase behavior on in situ viscosity, in which they found the molecular weight of polymer is the key factor dominating in situ rheology of semi-dilute polymers. Jouenne, et al. [41] performed degradation studies using a blender, capillaries and porous media. The kinetics of degradation fit a master curve as a function of normalized time, regardless of the media in which the degradation occurred. Moreover, the polymer will not be further degraded beyond the point of highest strain which determined the steady state value of degradation. Until the steady state was reached, degradation increased with the number of passes of the point of highest strain. This was reached at less than 6 mm in synthetic porous media. 

The present work aims to study the influence of mechanical degradation on HPAM rheology at a larger scale, at 5–10 cm of propagation. This was performed by flooding polymer through porous media and analyzing the injected and eluted samples. For this purpose, highly permeable outcrop rock (Bentheimer cores) was used. The experiments were carried out at room temperature using two types of semi-dilute HPAM polymers dissolved in brines with a given salinity. The polymers were pre-treated by prefiltering or pre-shearing through porous media prior to injection in order to represent the filtration and shearing processes induced on the polymer by the porous media in a field case. In such a field case, the polymer might be sheared near the injection well, where high flow velocities are achieved. High molecular weight fractions may be filtered by retention mechanisms as it propagates through the porous media at either high (near-well) or low velocity (deeper in the reservoir). Propagation effects were evaluated by re-injecting polymer that had already experienced high shear to evaluate if further mechanical degradation would occur at the same flow velocities (and thereby same shear rates). Results from this study reveal that pre-shearing at high flow rates (representative of near wellbore areas) has a larger impact than prefiltration. One consequence of this is that mechanical degradation due to high shear may improve injectivity without significantly reducing polymer in situ viscosity at (lower) reservoir flow rates. Another key observation is that polymer degradation occurs successively for high MW polymer. This indicates that it is not only the molecular weight and point of highest strain that determine the degree of degradation but also the exposure time and number of exposures to high strain. These are important aspects to implement in polymer flood design. 

## 2. Materials and Methods

### 2.1. Polymer Preparation 

Hydrolyzed polyacrylamide (HPAM) polymers with a 30% degree of hydrolysis were used for this study with different molecular weights and concentrations, as shown in Table 1. It is well-known that HPAM polymers have a broad spectrum of molecular weight distribution (e.g., Polydispersity Index > 1) [3,42]. These polymers on average contain 90% active material, as reported from the supplier. They were obtained as white granular powders from SNF Floerger, France. The polymer solutions were prepared by dissolving these polymers into 1 wt % NaCl brine. Initially, approximately 5000 ppm stock solution was carefully prepared and then diluted into the required concentrations. The preparation of stock solution was achieved by adding 3.0 g of polymer powder slowly into the shoulder of the vortex of 540.0 g brine while maintaining vigorous stirring using a magnetic stirrer until the vortex disappeared. Then, the stirring speed was decreased to 150 rpm and the polymer solution was left under slow mixing overnight. The prepared solution was kept at 5 °C inside a fridge and was used within two weeks of preparation.

The polymers were previously used in a different study and at the given concentrations and brine salinity, both polymers solutions A and B were within the semi-dilute region, see Skauge, et al. [23]. 

### 2.2. Brine

Brine solution containing 1 wt % NaCl was prepared by dissolving NaCl powder obtained from Sigma-Aldrich (Munich, Germany) into deionized water. Then the solution was filtered by using a 0.45 μm cellulose nitrate filter. The filtered brine was used in the preparation of polymer solutions and measurement of core petrophysical properties (e.g., porosity and permeability).

### 2.3. Porous Medium

Cylindrical cores of Bentheimer sandstone were used as porous medium with an average length and diameter of 10 and 3.8 cm, respectively. Also, short Bentheimer cores of 5 cm length were used for prefiltering and pre-shearing processes. Bentheimer sandstone is considered to be homogenous since it mainly contains quartz mineral [43]. The average core porosity and permeability for the 16 cores investigated were found to be 23% ± 1% and 2.4 ± 0.2 Darcy, respectively. The short cores were not measured directly, but were assumed to be within the range of the long cores reported in Table 2 and Tables 4–7. 

### 2.4. Rheology

Shear viscosity for polymer solutions was measured at 22 °C by using a Kinexus Pro (Malvern, UK) rheometer. Two types of geometries were used: cone-plate geometry (CP 2/50) with 2° inclination and 50 mm diameter in titanium was used for solutions with viscosity greater than 10 mPa.s, while a double gap geometry (DG 25) with 25 mm diameter was used for solutions with viscosity less than 10 mPa.s.

### 2.5. Core Flooding

**Figure 2:**
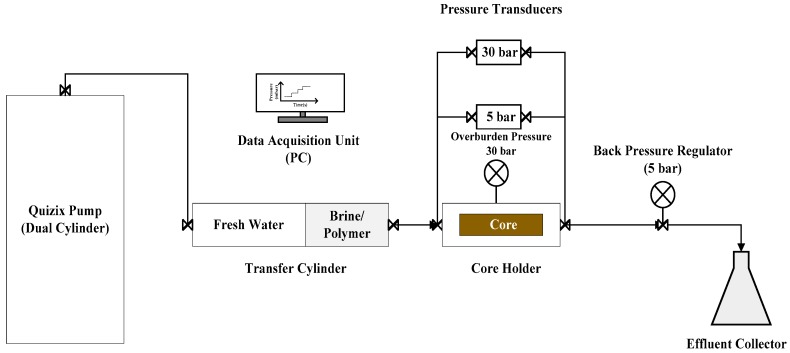
Schematic diagram of the core flooding apparatus.

The core flooding experiments were carried out under room temperature (22 °C). Figure 2 illustrates the experimental set-up which consists of dual piston Quizix pump, transfer cylinder, core holder, backpressure regulator and effluent collector, which were mounted in series. Differential pressure transducers with maximum range of 5 and 30 bar were mounted between the inlet and outlet of the core holder to monitor pressure drop across the core during injection. The backpressure regulator was mounted at the outlet of the core to apply a pressure of 5 bar to dissolve any air that maybe found within porous medium before flooding. The backpressure regulator was removed during polymer injection to avoid polymer degradation.

**Figure 3:**
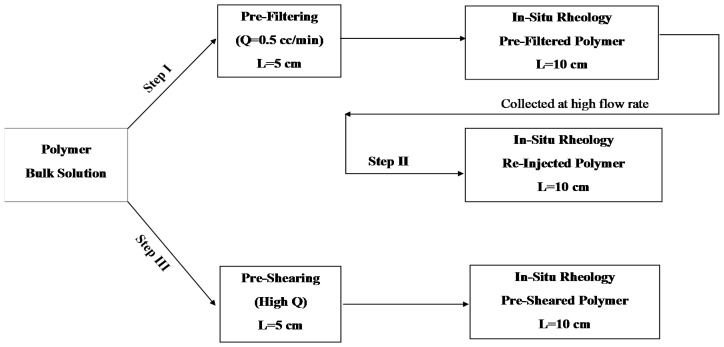
Flow chart of polymer pre-treatment methods and injection into porous medium.

Before injecting polymer solution into the cores for in situ rheology, the polymer solutions were pretreated differently as illustrated in Figure 3. Polymer pretreatment methods are described below:

- Step I, **pre-filtering** the polymer solution through short cores (*L* = 5 cm) at low flow rate (*Q* = 0.5 cc/min, $v_{D}=0.6\;m/\mathrm{day}$). The pre-filtered polymer solution will be then injected into longer core (*L* = 10 cm) for in situ rheology measurements. This step was carried out to avoid any microgel in the solution and filter out any possible large MW species. This step represents industrialized polymers which are used in field applications. It is also considered as a baseline for comparison with polymer solutions which were treated differently based on Steps II and III.
- Step II, **re-injecting** the effluent that was collected from Step I through a long core (*L* = 10 cm) at highest flow rate (e.g., the highest flow rate was achieved when the difference between overburden pressure and pump pressure is 10 bar). This injection rate was (*Q* = 12.0 and 15.0 cc/min, $v_{D}=15.5\;\mathrm{and}\;19.3\;m/\mathrm{day})$ for Solutions A and B, respectively. This step was carried out to investigate the effect of core length on the extent of degradation mechanisms as the degradation performed on long core compared to short core in Step III. Also, re-injected solution represents the flow of industrial polymer (prefiltered polymer) deep in reservoir that has already experienced filtration and degradation effects.
- Step III, **pre-shearing** the polymer solutions through short cores (*L* = 5 cm) at the highest flow rate obtained from Step II. Then, the pre-sheared solution is injected into a longer core (*L* = 10 cm) for in situ rheology measurements. In this step, large MW species in the solution are possibly filtered and mechanically sheared to lower MW species.

After saturating the core plugs with brine and porosity measurements, brine was injected into the cores at different flow rates to determine the absolute permeability. Absolute permeability ($K_{\mathrm{abs}}$) was obtained by Darcy’s law (Equation (1)):

(1) $$K_{\mathrm{abs}}=\frac{Q\cdot \eta \cdot L}{\Delta P\cdot A}$$

where, *Q* is injection flow rate, η is fluid viscosity, ΔP is pressure drop across the core, *L* and *A* are core length and cross-sectional area, respectively. Note that interstitial velocity (*v*) was obtained from Darcy velocity ($v_{D}$), where *ϕ* is the porosity of porous media.

(2) $$v=\frac{Q}{A\cdot \phi }=\frac{v_{D}}{\phi }$$

Polymer solution, after pretreatment, was injected into the core using Quizix pumps at low flow rate (*Q* = 0.5 cc/min, $v_{D}=0.6\;m/\mathrm{day}$) for at least 2 PV to satisfy porous medium polymer adsorption level and achieving stable differential pressure. Then, polymer flow rate was varied in a stepwise manner from the highest to the lowest rate. Each rate step was continued until a stabilized pressure drop across the core was achieved. Resistance factor (*RF*) was calculated as following [3]:

(3) $$RF=\frac{\Delta P_{p}}{\Delta P_{w}}$$

where $\Delta P_{p}$ is the pressure drop of polymer during polymer flow and $\Delta P_{w}$ is the pressure drop of brine before polymer flow in porous medium.

Samples of effluents were collected at different flow rates to measure their shear viscosity by rheometer and compared with initial solution viscosity. The following equation was used to express the change in shear viscosity by taking shear viscosity at shear rate of 10 s^−1^ [41]:

(4) $$\mathrm{Deg}\left(\%\right)=\frac{\eta_{i}-\eta_{e}}{\eta_{i-}\eta_{w}}100\%$$

where, $\eta_{i}$ is injected solution viscosity, $\eta_{e}$ is effluent viscosity and $\eta_{w}$ is brine viscosity which was measured to be 1.04 mPa.s.

After terminating the polymer injection, the core’s permeability to brine was re-measured after injecting 5 PV of brine at high flow rates proceeded by two steps of tapering. Tapering was performed by injecting diluted effluent at low flow rate (*Q* = 1.0 cc/min, $v_{D}=1.3\;m/\mathrm{day}$) with 50% and 25% of initial effluent concentration (e.g., in the case of Solution A, effluent of this polymer was collected and diluted into 250 and 125 ppm). Residual resistance factor (*RRF*), which is the permeability to brine before polymer flooding to permeability of brine after polymer flooding, was calculated using Equation (5) [44]:

(5) $$RRF=\frac{K_{\mathrm{wi}}}{K_{\mathrm{wf}}}$$

where $K_{\mathrm{wi}}$ is the absolute permeability to brine before polymer flow and $K_{\mathrm{wf}}$ is the absolute permeability to brine after polymer flow after tapering.

## 3. Results and Discussion

### 3.1. Shear Viscosity 

**Figure 4:**
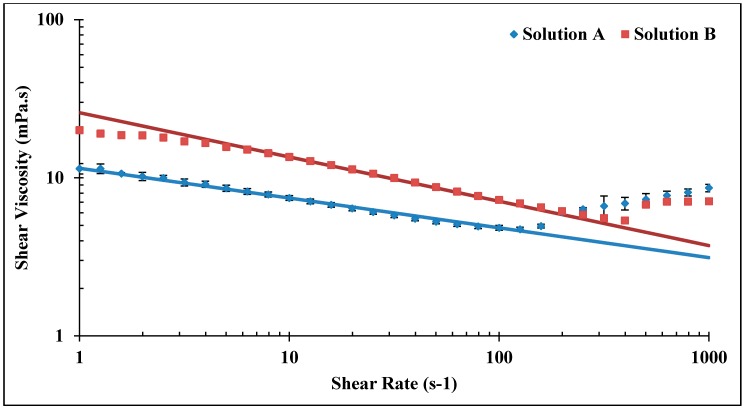
Shear viscosity of bulk Solutions A and B in 1 wt % NaCl at 22 °C. Solid lines represent power law model. Error bars for Solution B are smaller than the size of each point (±0.20 mPa.s).

Shear viscosity measurements were carried out for shear rates 1–1000 s^−1^ as shown in Figure 4. Rheometer measurements were very well matching with the power law model by setting *n* = 0.81 and 0.72 and *k* = 11.49 and 25.80 for bulk Solutions A and B, respectively. Both solutions show predominantly shear thinning behavior, while Solution B shows indications of a Newtonian plateau for shear rates <2 s^−1^. The viscosity increase observed at high shear rates is mainly due to turbulence flow caused by high rotational speed (which is also observed for brine) that causes an artifact in measurements [45]. The turbulence should not be confused with the apparent shear thickening observed in porous media flow. Shear viscosity is 7.45 and 13.54 mPa.s for bulk Solutions A and B, respectively, using the reservoir flow relevant shear rate of 10 s^−1^ as a reference.

### 3.2. Apparent Viscosity in Porous Medium vs. Shear Viscosity in Rheometer 

**Figure 5:**
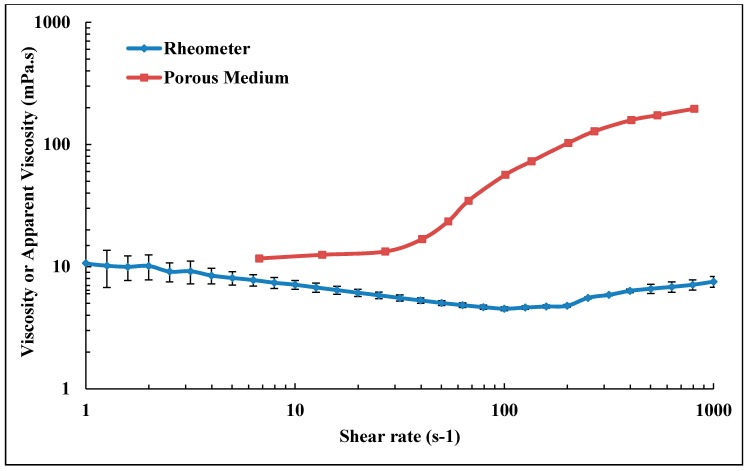
Viscosity of pre-filtered Solution A (Concentration = 500 ppm, MW = 18 MDa) measured in porous medium and in bulk.

In order to compare the flow of polymer in porous medium with the flow in rheometer, it is necessary to translate the flow velocity in porous medium to shear rate. Determining the exact equivalent shear rate in porous medium is not possible due to its distribution of pore size, tortuosity and complexity. The following equation is conventionally used to estimate an effective shear rate in porous medium [7]:

(6) $$\dot{\gamma }=\alpha \frac{4v_{D}}{\sqrt{8K_{w}\phi }}$$

where $v_{D}$ is Darcy fluid velocity, *ϕ* porosity, $K_{w}$ absolute permeability, *α* shape factor, which is an empirical parameter. Here we have applied an *α* value of 2.5 for consolidated sandstone (Bentheimer) [3]. The apparent viscosity of polymer flowing in porous medium is represented by the resistance factor (*RF*). Given that the other factors in Equation (1) are the same, apparent viscosity equals RF × μ_w_. Apparent viscosity was calculated from Equation (3) for each flow rate. The comparison of the shear viscosity from the rheometer and apparent viscosity from the porous media for Solution A is shown in Figure 5. At lower shear rates ($\dot{\gamma }$ < 30 $s^{-1}$), apparent viscosity approaches the upper Newtonian plateau observed from bulk flow shear viscosity. However, at moderate and high shear rates, apparent viscosity in porous medium diverges from viscosity measured in rheometer. This is clearly seen in that Newtonian and shear thickening behaviors are observed in porous medium, while shear thinning behavior is shown by rheometer. However, the two curves are not expected to show the same trend as flow in porous medium is different from shear flow in a rheometer.

### 3.3. Apparent Viscosity 

**Figure 6:**
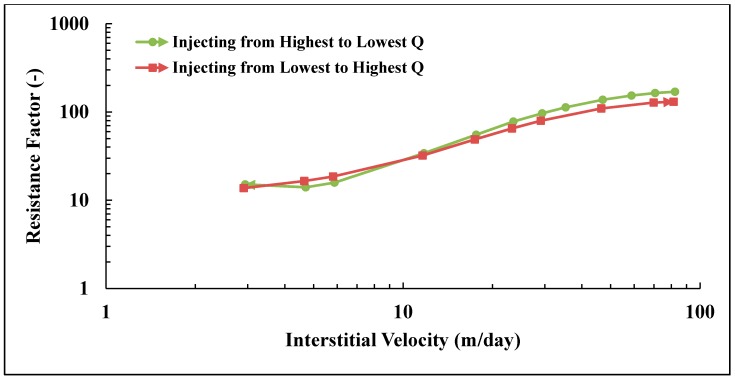
Resistance factor versus interstitial velocity for pre-sheared Solution A. The polymer was sheared at *Q* = 12 cc/min and RF measured at different injection schemes.

The apparent viscosity of polymer flowing in porous medium is represented by the resistance factor (*RF*), see Figure 6. *RF* was calculated from Equation (3) for each flow rate and is plotted vs. interstitial velocity as calculated from Equation (2). Steady state conditions were achieved at each flow rate before changing to the next rate. The experiment was performed on twin plugs (see Table 2) with the same polymer. An apparent Newtonian plateau is observed at low flow velocity. Above a critical flow velocity, i.e., 3–5 m/day, shear thickening is observed. It was found that the injection scheme from lowest to highest flow rate has lower RF at higher velocities; the difference between the schemes was 23% at the highest velocity. The difference is relatively smaller at lower velocities. The pressure was stable for the highest rate for both schemes and the reason for the discrepancy is not clear. It is, however, reproducible and may be due to difference in hydrodynamic retention for a core saturated at high rate versus one saturated at a low rate. Since adsorption measurements were not performed for the two cases, no firm conclusions can be made on this matter. The injection scheme from highest to lowest flow rates was adopted for this study. This may also be more representative for flow velocities experienced in a reservoir where it is subjected to high velocities in near wellbore areas.

### 3.4. Backpressure Regulator Effects

**Figure 7:**
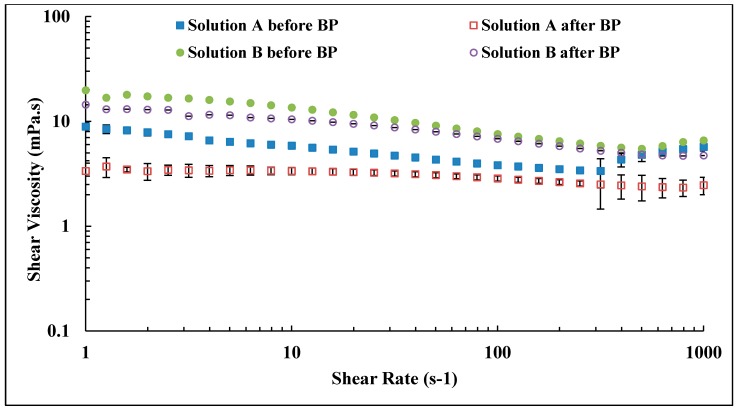
Shear viscosity vs. shear rate of polymers injected through backpressure regulator at high flow rates.

Typical core flood setup consists of a backpressure regulator which is used to stabilize the pressure across the core and removes any air within the system. However, it was found that the backpressure regulator can induce a mechanical degradation of polymer, as can be seen from the reduction of shear viscosity as presented in Figure 7 and the tabulated data in Table 3. High molecular weight polymer with low concentration (Solution A) experience high degradation compared to lower molecular weight polymer (Solution B). Solution A lost more than 50% of its original viscosity after passing backpressure regulator at high flow rates. The flow rates applied for the investigation were *Q* = 12 and 15 cc/min for Solutions A and B, respectively. Please note that the backpressure regulator was not used during investigation of polymer in situ rheology to avoid mechanical degradation of polymer.

### 3.5. In Situ Viscosity of Solution A

**Figure 8:**
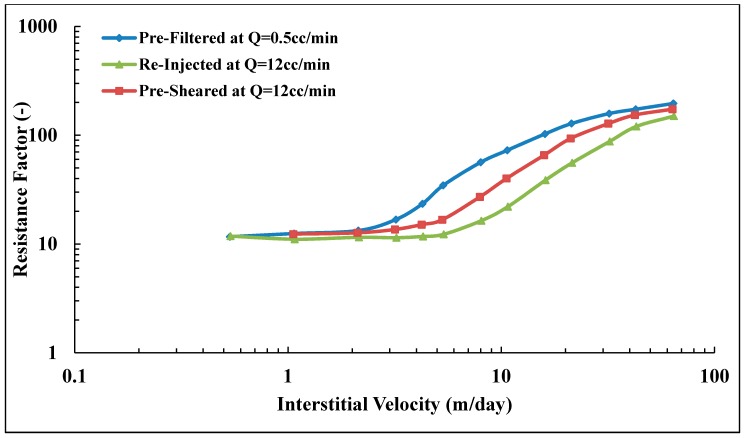
Resistance factor (*RF*) vs. interstitial velocity for Solution A.

Three core floods were performed to determine the influence of polymer pre-treatment on in situ viscosity. The three pre-treatment methods are described in Figure 3. Core data are given in Table 4. Resistance factor as a function of flow velocity for Solution A (Concentration = 500 ppm, MW = 18 MDa) is presented in Figure 8. All three core floods show apparent Newtonian behavior at low flow velocities followed by shear thickening behavior at higher flow velocities. At low flow velocities, all the solutions approach an *RF* value of ~12 regardless of their pre-treatment procedure. However, after the onset of shear thickening, the solutions show distinctly different *RF* curves. The pre-filtered solution exhibits the highest *RF* compared to the pre-sheared and reinjected solutions. The *RF* correlates to the onset of shear thickening. Pre-filtered solution has an onset of shear thickening at $v_{c}$ = 2.5 m/day, which is lower than for pre-sheared and reinjected solutions which have *v*_c_ = 4.0 and 6.7 m/day, respectively. The onset of shear thickening was measured apparently from the *RF* curve that represents the point of departure from Newtonian to shear thickening behavior. The shift in the onset of shear thickening to higher velocities indicates that the polymer has experienced degradation and that the molecular weight distribution is altered [25,26].

In addition to the effect on the onset of shear thickening, mechanical degradation markedly affects the degree of shear thickening. The degree of shear thickening is represented by the slope of shear thickening part of the *RF* curve, i.e., the change of RF with respect to flow velocity. The higher slope was found for pre-filtered solution, *m* = 7.7 ${\left(m/\mathrm{day}\right)}^{-1}$ compared to pre-sheared and reinjected solutions (*m* = 4.3 and 3.0 ${\left(m/\mathrm{day}\right)}^{-1}$, respectively). Reinjected solution experienced further degradation when reinjected into porous media. This indicates further degradation occurred with increasing core length.

**Figure 9:**
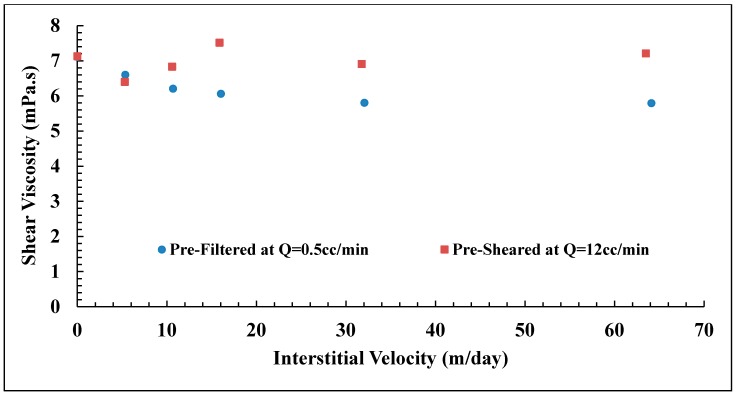
Effluents shear viscosity of Solution A measured at $\dot{\gamma }$ = 10 $s^{-1}$. Error bars are smaller than the size of each point (±0.20 mPa.s).

During the polymer injection, shear viscosity of effluents were measured as shown in Figure 9. The viscosity at *v* = 0 m/day means the injected viscosity of solution after passing pre-treatment processes ($\eta_{i}$). The highest flow velocity (*v* = 64.1 m/day) for the pre-filtered solution showed a shear degradation of 22%. However, no significant shear degradation was observed for pre-sheared solutions. The injected viscosity of the reinjected solution is lower than that of injected pre-filtered and pre-sheared solutions which could explain the lower *RF* observed in the porous medium. Still, this is not sufficient to explain the difference in *RF* curves observed for pre-sheared and pre-filtered solutions that have similar injected viscosity but show different flow behavior in the porous medium. This is further evidence that in situ viscosity cannot be predicted from bulk shear viscosity measurements, even for flooding experiments with the same polymer, concentration, brine and temperature. However, this is only true for flow velocities above the onset of shear thickening. Below, at typical reservoir flow velocities, the in situ viscosities are very similar. The pre-treatment at high shear reduces the extensional viscosity [25,26,46,47,48]. This confirms that the high *RF* values at high flow velocities are due to extensional viscosity that is negligible at low velocities in which shear viscosity is more dominant. 

### 3.6. In Situ Viscosity of Solution B

**Figure 10:**
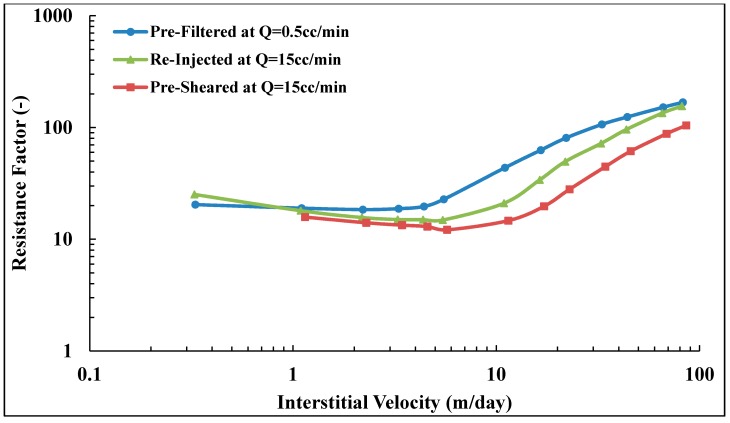
Resistance factor (*RF*) versus interstitial velocity for Solution B (Concentration = 1000 ppm, MW = 12 MDa).

The same procedures were applied for Solution B (Concentration = 1000 ppm, MW = 12 MDa), which has a shear viscosity of 13.54 mPa.s at 10 $s^{-1}$. Figure 10 shows *RF* curves for Solution B at different preparation procedures. Core data are given in Table 5. At low flow velocities, a weak shear thinning behavior was observed, which was not observed for Solution A. This is most likely because Solution B has a higher concentration than polymer A, for which the degree of entanglements is higher in Solution B. Another observation is that all three solutions approach similar *RF* values at low velocities, which shows that the degree of pre-shearing and re-injection of polymer at high rates do not significantly change the in situ viscosity at typical reservoir flow rates. This is in sharp contrast to higher flow rates, where significant differences are observed. It was found that the pre-filtered solution possesses an earlier onset of shear thickening ($v_{c}$ = 4.1 m/day) compared with pre-sheared and reinjected solutions ($v_{c}$ = 12.0 and 7.7 m/day; respectively). Furthermore, the degree of shear thickening was higher for pre-filtered solution, *m* = 3.5 ${\left(m/\mathrm{day}\right)}^{-1}$ compared to pre-sheared and reinjected solutions (*m* = 1.5 and 2.3 ${\left(m/\mathrm{day}\right)}^{-1}$, respectively). 

**Figure 11:**
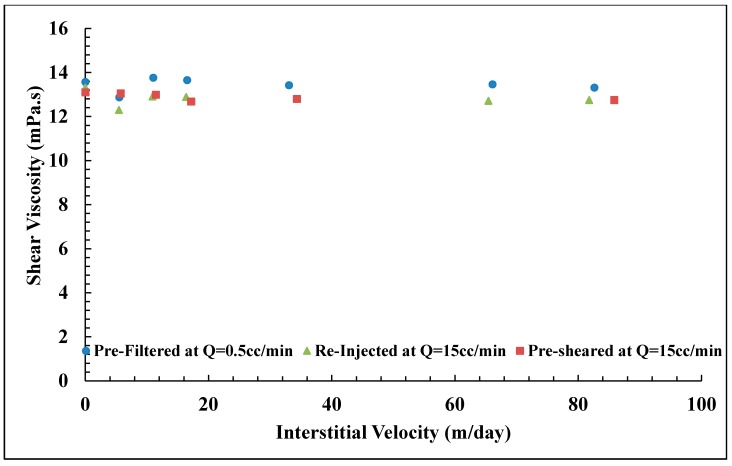
Effluent shear viscosity of Solution B measured at $\dot{\gamma }$ = 10 $s^{-1}$. Error bars are smaller than the size of each point (±0.20 mPa.s).

Effluent viscosities do not show significant degradation for any of the three core floods as shown in Figure 11. This corresponds well with the *RF* curves that show similar viscosity at typical reservoir flow velocities, i.e., 0.1 to 1.0 m/day. However, the effluents collected at higher flow velocities, such as 30 to 70 m/day also show similar values, while the *RF* values are significantly different. This shows that the difference in shear thickening at high velocities is due to extensional viscosity, which is not reflected in the measurements of shear viscosity. 

### 3.7. Influence of Degradation on Polymer Molecular Weight

**Figure 12:**
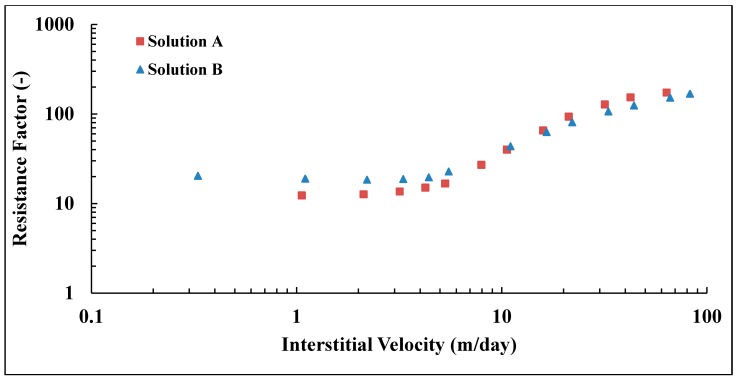
Influence of pre-shearing on Solution A (MW = 18 MDa) compared to pre-filtered Solution B (MW = 12 MDa).

Figure 12 shows the effect of pre-shearing on the onset of shear thickening for high molecular weight polymer (Solution A) to that of lower molecular weight polymer (Solution B). Shift in onset of shear thickening has been an indication of reduction of molecular weight distribution [9,23,25]. It is generally difficult to quantify the reduction of MWD due to the difficulties in determining MW of high molecular weight polymers by methods such as size exclusion chromatography (SEC) and asymmetric flow field-flow fractionation (AF4) [49,50,51,52]. To characterize the reduction by core floods would usually require a large number of core floods. In this experiment, the onset of shear thickening and the shape of the *RF* curves are similar after the onset of shear thickening for pre-sheared Solution A and pre-filtered Solution B (Figure 12). This indicates the MWD of Solution A was shifted to lower distribution similar to that of prefiltered Solution B. This supports the observation given by Puls, et al. [53], in which the pre-shearing process reduces HPAM molecular weight distribution, as they observed by using size exclusion chromatography (SEC) for determining the MW of solution. 

### 3.8. Permeability Reduction

HPAM flowing in a porous medium will adsorb on rock surfaces and be trapped within narrow pores, resulting in polymer retention. Polymer retention consequently causes permeability reduction which can be experimentally evaluated by residual resistance factor (*RRF*). Lake [2] reported that *RRF* can be reduced if the polymer is pre-sheared before injection. *RRF* is a function of polymer molecular weight, degree of hydrolysis, flow velocity and pores structures. Other authors, such as Yerramill, et al. [54] and Morris and Jackson [46], reported that *RRF* increases with increasing polymer concentration. 

**Figure 13:**
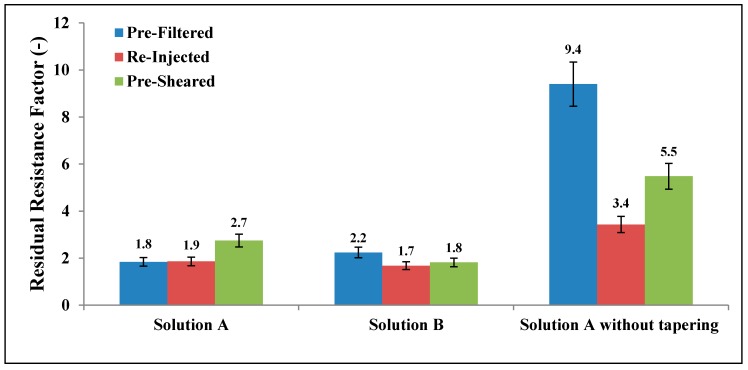
Residual resistance factor (*RRF*) after tapering for both solutions and without tapering for Solution A.

*RRF* values obtained for Solutions A and B in this experimental study are shown in Figure 13. The *RRF* for pre-filtered Solutions A and B were 1.8 and 2.2, respectively. In this context, the difference in *RRF* is regarded as small, particularly when considering the difference in concentration and molecular weight for Solutions A and B (Concentration = 500 and 1000 ppm, MW = 18 and 12 MDa, respectively). Similarly, there were small differences in *RRF* between degraded (pre-sheared and re-injected) and non-degraded solutions (pre-filtered). 

How to measure “true” *RRF* has been debated [55]. In this experiment, the influence of tapering on *RRF* was quantified. *RRF* values were reduced from 9.4 to 1.8 for pre-filtered Solution A before and after tapering; respectively. Recall that tapering was performed to flush out as much as possible of the retained polymer. Tapering was performed in two steps before water post-flush by injecting diluted polymer effluent with 50 and 25% of initial effluent polymer concentration. The error bars in Figure 13 are based on repeated measurements. 

### 3.9. Reproducibility of Experiments

The shift in onset of shear thickening to higher velocities and the reduction in slope for pre-sheared and re-injected solutions are consistent with a mechanical degradation and alteration of polymer molecular weight distribution. Figure 8 shows a successive degradation of high molecular weight polymer as it is reinjected into porous media, while this is in contrast with the lower molecular weight solution shown in Figure 10. It was not clear if the polymer might be exposed to further degradation during reinjection, as reported by Sorbie and Roberts [56] and Al Hashmi, et al. [31].

**Figure 14:**
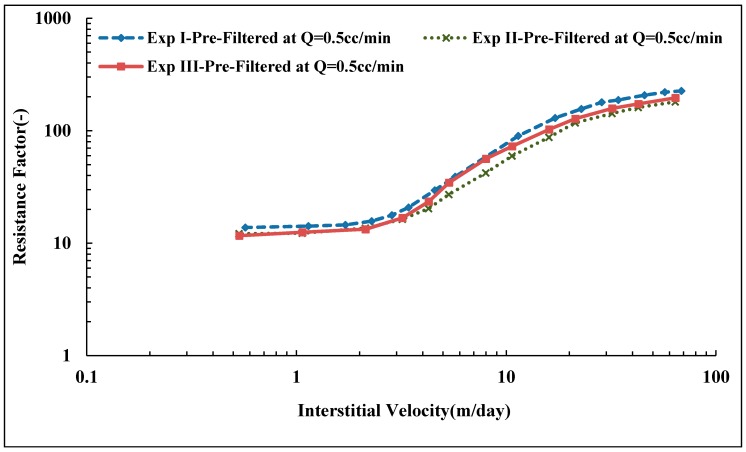
Reproduced resistance factor vs. interstitial velocity of pre-filtered Solution A.

To further investigate the difference on the extent of mechanical degradation found between pre-shearing and reinjection in which the core length has been varied. Similar sets of experiments were preformed, which confirm that resistance factor of prefiltered solutions for both polymers are reproducible, as shown in Figure 14. This confirms that RF is reproducible for the prefiltered solution, which indicates the cores were fairly homogenous in that they have quite similar absolute properties (e.g., porosity and permeability as shown in Table 6). 

**Figure 15:**
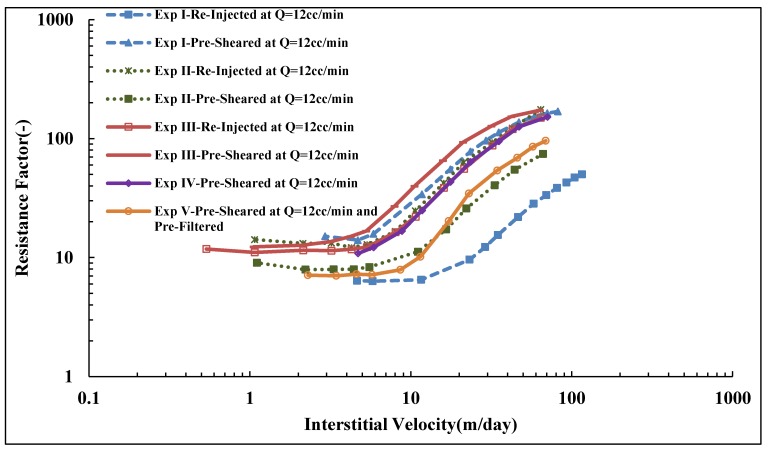
Reproduced resistance factor vs. interstitial velocity of pre-sheared and reinjected Solution A.

However, some disparity was found on reproducing resistance factor of pre-sheared and reinjected solutions. It was found in some experiments that reinjected solution experienced further degradation compared to pre-sheared solution, as in Exp I and III in Figure 15. The high degradation found of reinjected solution on Exp I is attributed to the associated effect with the use of the backpressure regulator, which was not used in the rest of the experiments. The match between RF curves of pre-sheared solution in Exp II and V demonstrates that the pre-shearing process can filter and pre-shear the polymer. A disparity was found in reproducing the resistance factor of the pre-sheared solution as well. This could be expected due to the fact that pretreated polymer could experience different fields of shear rate due to different topology of the cores, regardless of the similarity between the cores’ apparent petrophysical properties (see Table 6). The pre-shearing process could further or similarly degrade the polymer as the reinjection process does, and vice versa. This indicates that the core length or exposure time has an effect on the degradation mechanism, although some previous studies [41] have reported that polymer degradation has less dependency in on core length. This could be true in synthetic porous medium but not in realistic porous media such core plugs. However, the dependence of successive polymer degradation on characteristic length to approach a steady state value in a realistic porous medium is ambiguous and still not defined. 

### 3.10. Pre-Shearing at Very High Flow Rates

**Figure 16:**
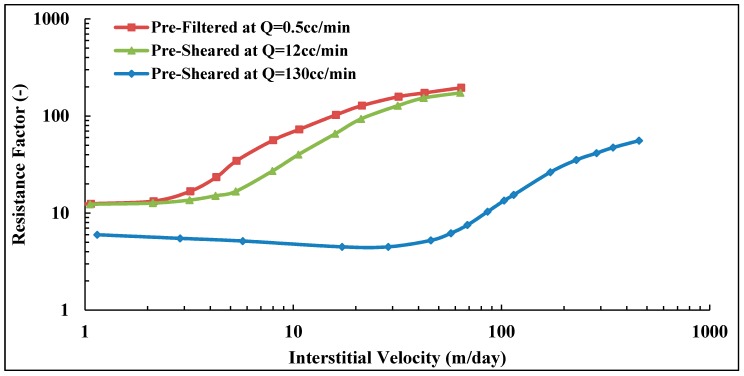
Resistance factor of pre-sheared Solution A at different flow rates verses prefiltered solution.

The pre-shearing process described in Figure 3 was based on the highest shear rate that was achieved from the first step which was limited by experimental setup. In this experiment, the polymer (Solution A) was pre-sheared at extremely high flow rate (*Q* = 130 cc/min, $v_{D}$ = 166.8 m/day) in a short core and its shear viscosity degraded by 32% compared to original bulk viscosity of 6.16 mPa.s. It can be seen in Figure 16 that a significant reduction in its in situ viscosity, i.e., the reduction is more than 50% compared to prefiltered and pre-sheared polymers at lower flow rate. Moreover, a significant alteration can be observed on viscoelastic properties of pre-sheared polymer at high flow rate. The onset of shear thickening is shifted to much higher velocities and the degree of shear thickening reduced significantly. Furthermore, it was found that residual resistance factor can be reduced if the polymer is submitted to high degradation as can be seen in Table 7. The core properties of prefiltered and pre-sheared polymer at *Q* = 12 cc/min were presented earlier in Table 4.

**Figure 17:**
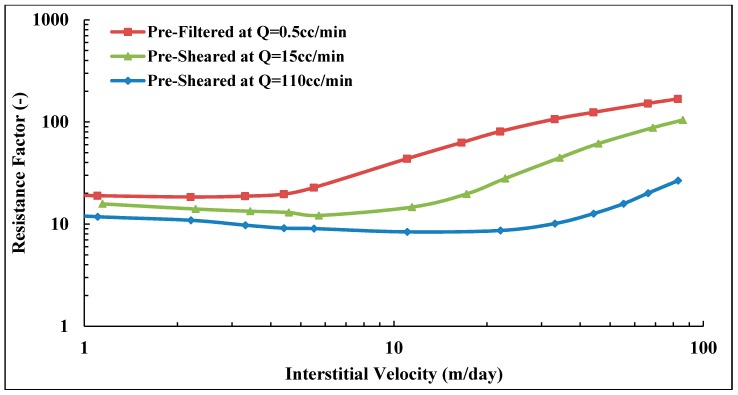
Resistance factor of pre-sheared Solution B at different flow rates versus prefiltered solution.

Similar observations were found for Solution B with lower molecular weight that was pre-sheared at high flow rate (*Q* = 110 cc/min, $v_{D}$ = 141.2 m/day). Please note that the flow rate applied for pre-shearing this solution was lower than Solution A due to the higher concentration of Solution B. Its shear viscosity degraded by 16% compared to the bulk viscosity of Solution B, which was 13.25 mPa.s. This results in a significant loss of its in situ viscosity, as well, compared to prefiltered and pre-sheared solutions at lower flow rates, as can be seen in Figure 17. Its viscoelastic parameters reduced, and the onset of shear thickening shifted to much higher velocities, while the degree of shear thickening is reduced. Core properties and viscoelastic parameters for prefiltered and pre-sheared Solution B at low and high flow rates are given in Table 5 and Table 7, respectively.

It can be also seen from Figure 16 and Figure 17 that both solutions responded differently to the high flow rate applied for each of them. High molecular weight polymer suffers more degradation compared to lower solution as discussed earlier in this paper. Although it was pre-sheared at a lower rate compared to Solution B. Hence, the applied flow rate for the pre-shearing process has to be optimized to avoid the loss of polymer viscosity while improve its viscoelastic properties that results in better injectivity.

## 4. Conclusions

The influence of mechanical degradation and filtration on in situ rheology has been investigated for two HPAM polymers with different molecular weights. Three conditions were evaluated: (1) solutions filtered through a short Bentheimer core at low flow rate (prefiltering); (2) prefiltered solutions subsequently injected at high rate through a Bentheimer core (reinjected); and (3) solutions mechanically degraded through a short Bentheimer core at high flow rate (pre-shearing). The following conclusions could be made:

- At high flow velocities, similar to those experienced in the near wellbore area of an injector, polymer flow history plays a substantial role for HPAM in situ viscosity. Solutions exposed to high rates were mechanically degraded and showed delay in onset of shear thickening and reduction in apparent viscosity compared to the solutions exposed to low rates.
- All solutions, regardless of previous exposure to high or low rates, showed similar apparent viscosity and predominantly Newtonian behavior at low velocities (i.e., reservoir velocities).
- These results show that mechanical degradation is beneficial for the polymer types and concentrations investigated here since injection pressures are reduced and reservoir apparent viscosities are maintained.
- Polymer flow history (pretreatment) has little impact on residual resistance factors (*RRF*). *RRF* is found to be more influenced by fluid exchange process (tapering).
- Mechanical degradation during polymer reinjection is coupled to several parameters such as characteristics of the porous media, flow rate, geometry (inlet/outlet effect), and polymer exposure time to the porous media. Results indicate that re-exposure to the same shear conditions (same flow rate and porous media) may lead to additional mechanical degradation.
- HPAM flowing in porous medium at low velocities (e.g., reservoir velocities) show predominantly Newtonian behavior followed by shear thickening at higher flow velocities (e.g., at wellbore area). Both flow behaviors are absent in rheometer measurements that demonstrate a predominantly shear thinning behavior at comparable flow rates. This conclusion is limited to the polymer concentration used (weak semi-dilute region).
- Initial studies show that a backpressure regulator can induce a mechanical degradation of polymer that reduces its viscoelastic properties and may lead to erroneous conclusions from laboratory experiments. All experiments reported were made without a backpressure regulator.

## Figures and Tables

**Table 1:** Physicochemical properties of polymers.

Solution	Polymer (Flopaam) Type	Molecular Weight ($10^{6}$ g/mol = MDa)	Polymer Concentration (mg/L = ppm)	Viscosity (mPa.s)
A	3630 s	18	500	7.45
B	3430 s	12	1000	13.54

**Table 2:** Properties of cores used for hysteresis investigation of polymer injection scheme.

Injection Scheme	*L* (cm)	*D* (cm)	*ϕ* (-)	*K*_wi_ (Darcy)
Injection from lowest to highest *Q*	9.41	3.75	0.22	2.17
Injection from highest to lowest *Q*	9.44	3.78	0.22	2.18

**Table 3:** Effect of backpressure regulator on shear viscosity of Solutions A and B. Shear viscosity measured at $\dot{\gamma }$ = 10 $s^{-1}$.

Measurements	Solution A		Solution B	
	Before BP	After BP	Before BP	After BP
Shear Viscosity (mPa.s)	5.85	3.36	13.53	10.43
Deg (%)	0.00	51.8	0.00	24.8

**Table 4:** Core and solution properties for injected Solution A (Concentration = 500 ppm, MW = 18 MDa).

Exp.	*L* (cm)	*D* (cm)	*ϕ* (-)	*K*_wi_ (Darcy)	*K*_wf_ (Darcy)	*RRF* (-)	$\eta_{i}$ (mPa.s)	$\eta_{e}$ (mPa.s)	*v*_c_ (m/day)	*m* (m/day)^−1^
Pre-filtered at *Q* = 0.5 cc/min	9.82	3.77	0.24	2.57	1.40	1.84	7.11	5.79	2.51	7.68
Re-injected at *Q* = 12 cc/min	9.78	3.77	0.24	2.39	1.28	1.86	5.79	-	6.71	3.00
Pre-sheared at *Q* = 12 cc/min	9.72	3.77	0.24	2.25	0.82	2.75	7.13	7.21	4.00	4.32

**Table 5:** Core and solution properties for injected Solution B (Concentration = 1000 ppm, MW = 12 MDa).

Exp.	*L* (cm)	*D* (cm)	*ϕ* (-)	*K*_wi_ (Darcy)	*K*_wf_ (Darcy)	*RRF* (-)	$\eta_{i}$ (mPa.s)	$\eta_{e}$ (mPa.s)	*v*_c_ (m/day)	*m* (m/day)^−1^
Pre-filtered at (*Q* = 0.5 cc/min)	9.82	3.79	0.23	2.16	0.96	2.24	13.57	13.31	4.06	3.50
Re-injected at (*Q* = 15 cc/min)	9.57	3.79	0.23	2.08	1.24	1.68	13.31	12.75	7.69	2.28
Pre-sheared at (*Q* = 15 cc/min)	10.27	3.77	0.23	2.80	1.54	1.82	13.10	12.75	11.99	1.46

**Table 6:** Core and solution properties for reproducibility study performed with Solution A (Concentration = 500 ppm, MW = 18 MDa).

Exp.	*L* (cm)	*D* (cm)	*ϕ* (-)	*K*_wi_ (Darcy)	*K*_wf_ (Darcy)	*RRF* (-)	$\eta_{i}$ (mPa.s)	$\eta_{e}$ (mPa.s)
Exp I-Pre-Filtered at *Q* = 0.5 cc/min	9.37	3.76	0.23	2.59	0.28	9.4	7.34	5.26
Exp I- Re-Injected at *Q* = 12 cc/min	9.58	3.77	0.22	2.28	0.67	3.4	5.26	4.84
Exp I- Pre-Sheared at *Q* = 12 cc/min	9.44	3.78	0.22	2.18	0.40	5.5	-	-
Exp II- Pre-Filtered at *Q* = 0.5 cc/min	9.54	3.77	0.24	2.48	1.35	1.8	6.81	6.62
Exp II- Re-Injected at *Q* = 12 cc/min	9.81	3.77	0.24	2.41	1.17	2.1	6.62	6.18
Exp II- Pre-Sheared at *Q* = 12 cc/min	9.74	3.77	0.23	2.19	0.98	2.2	4.06	4.55
Exp III- Pre-Filtered at *Q* = 0.5 cc/min	9.82	3.77	0.24	2.57	1.40	1.8	7.11	5.79
Exp III-Re-Injected at *Q* = 12 cc/min	9.78	3.77	0.24	2.39	1.28	1.9	5.79	-
Exp III-Pre-Sheared at *Q* = 12 cc/min	9.72	3.77	0.24	2.25	0.82	2.8	7.13	7.21
Exp IV -Pre-Sheared at *Q* = 12 cc/min	9.88	3.78	0.22	2.31	1.12	2.1	6.51	6.30
Exp V-Pre-Sheared at *Q* = 12 cc/min and Pre-Filtered at *Q* = 0.5 cc/min	10.00	3.79	0.22	2.72	1.15	2.4	5.93	5.37

**Table 7:** Core and solution properties for pre-sheared Solution A and B at very high flow rates.

Exp.	*L* (cm)	*D* (cm)	*ϕ* (-)	*K*_wi_ (Darcy)	*K*_wf_ (Darcy)	*RRF* (-)	$\eta_{i}$ (mPa.s)	$\eta_{e}$ (mPa.s)	*v*_c_ (m/day)	*m* (m/day)^−1^
**Solution A**	8.87	3.78	0.22	2.37	1.49	1.6	4.52	4.47	53.61	0.18
Pre-sheared at *Q* = 130 cc/min										
**Solution B**	9.71	3.79	0.23	1.87	1.09	1.7	11.30	11.26	33.5	0.37
Pre-sheared at *Q* = 110 cc/min										

## Nomenclature

*A*	cross-sectional area (cm^2^)
AF4	Asymmetrical flow field-flow fractionation
*BP*	backpressure
*C**	critical overlap concentration (ppm)
*D*	core dimeter (cm)
EOR	enhanced oil recovery
HPAM	partially hydrolyzed polyacrylamide
*k*	viscosity constant
$K_{\mathrm{abs}}$	absolute permeability to brine (Darcy)
*K*_wf_	absolute permeability to brine after polymer flow (Darcy)
*K*_wi_	absolute permeability to brine before polymer flow (Darcy)
*L*	core length (cm)
MW	molecular weight (MDa)
MWD	molecular weight distribution, dimensionless
*n*	power law index, dimensionless
PV	pore volume, dimensionless
*Q*	flow rate (cc/min)
*RF*	resistance factor, dimensionless
*RRF*	residual resistance factor, dimensionless
SEC	size exclusion chromatography
*v*	interstitial velocity (m/day)
$v_{c}$	onset of shear thickening (m/day)
$v_{D}$	Darcy velocity (m/day)
$\Delta P_{p}$	pressure drop during polymer flow (bar)
$\Delta P_{w}$	pressure drop during water flow (bar)
$\eta_{e}$	effluent viscosity (mPa.s)
$\eta_{i}$	injected solution viscosity(mPa.s)
$\eta_{w}$	brine viscosity (mPa.s)
*ϕ*	porosity, dimensionless
$\dot{\gamma }$	shear rate ($s^{-1}$)
